# Supplementary material for: Turkish validation of a resilience scale from older people’s perspectives: resilience scale for older adults
Source: PeerJ. 2025 Jan 21;13:e18837. doi: 10.7717/peerj.18837 (PMC11758914; doi:10.7717/peerj.18837)
Supplement: Supplemental Information 1 [file peerj-13-18837-s001.docx]

# SOCIODEMOGRAPHIC FORM

# Gender: Female/Male

# Age: ….

# Marital Status: Married/Widowed/Single

# Level of Education: Primary School /Middle School/ High School/ University

# Residential Situation: Alone /In a house with relative or spouse/Nursing Home/With a caregiver

# Income Status: My income is less than my expenses/ My income is equal to my expenses/ My income is more than my expenses

# Employment Status: Retired /Currently employed(Part-time/Full-time)/ Never employed

# Occupation: ….

# Having a Chronic Diseases: Yes/No

# Number of Chronic Diseases: …

# Names of Chronic Diseases: …

# Number of Drugs sed in a day: …

# Sleep Quality: Good/ Moderate/Poor

**Resilience Scale for Older Adults**

*Instructions:* Below are a list of statements that you may agree or disagree with. Please indicate your agreement with each item using the 1-5 scale below. There are no right or wrong answers so please be open and honest.

| **Strongly Disagree** | **Disagree** | **Neither Agree nor Disagree** | **Agree** | **Strongly Agree** |
| --- | --- | --- | --- | --- |
| **1** | **2** | **3** | **4** | **5** |

1. When faced with challenges I am persistent.
2. I am determined to achieve my goals.
3. I will not give up on something just because it is difficult.
4. When I put my mind to a task, I can effectively complete it.
5. I am capable of achieving my goals.
6. I can accomplish things on my own.
7. I believe things will usually work out in the end.
8. I try to make the most out of any situation.
9. I have a positive attitude towards most things.
10. I understand what makes my life meaningful.
11. I try to live life to the fullest.
12. I try to live each day as if it were my last.
13. I feel like I belong to something.
14. I am not alone.
15. People would miss me if I went away.
16. I have family members I can rely on.
17. I can ask my family for help if something bad happens.
18. I feel important to my family.
19. My friends are there for me when I need them.
20. My friends are important sources of support for me.
21. My neighbours will help me when I need it.
22. I pray to help me through hard times.
23. Praying to God helps me cope when something bad happens.
24. I pray regularly.
25. When life gets hard, I place my trust in my god.
26. I believe God is watching over me.
27. I believe God will not give me more than I can handle.
28. I have learned a lot from my past experiences.
29. I have faced adversity in my life.
30. The challenges in my life have taught me valuable lessons.
31. It is important to take care of yourself.
32. I try to prevent bad things from happening.
33. I try to be proactive when faced with challenges.

# Perceived Stress Scale 4 (PSS-4)

Instructions: The questions in this scale ask you about your feelings and thoughts during THE LAST MONTH. In each case, please indicate your response by selecting the option representing HOW OFTEN you felt or thought a certain way.

Never; Almost never; Sometimes; Fairly often; Very often

1. In the last month, how often have you felt that you were unable to control the important things in your life?
2. In the last month, how often have you felt confident about your ability to handle your personal problems?
3. In the last month, how often have you felt that things were going your way?
4. In the last month, how often have you felt difficulties were piling up so high that you could not overcome them?

**Older people’s quality of life-brief (OPQOL-brief)**

# OPQOL-brief

**Notes:**

The OPQOL-BRIEF questionnaire has 13 items, with a preliminary single item on global QoL, shown below. This single item is not scored with the OPQOL; it is coded as Very good (1) to Very bad (5).

**OPQOL-Brief scoring:**

Each of the 13 items is scored Strongly agree=1, Agree=2, Neither=3, Disagree=4, Strongly disagree=5. The items are summed for a total OPQOL-Brief score, then positive items are reverse coded, so that higher scores represented higher QoL.

**We would like to ask you about your quality of life:**

Single item - global QoL:

# Thinking about both the good and bad things that make up your quality of life, how would you rate the quality of your life as a whole?

Your quality of life

as a whole is: Very good Good Alright Bad Very bad

# OPQOL-Brief

1. **Please tick one box in each row. Please select the response that best describes you/your views. There are no right or wrong answers.**

Strongly Agree Neither Disagree Strongly agree agree nor disagree

disagree

1. I enjoy my life overall
2. I look forward to things
3. I am healthy enough to get out and about
4. My family, friends or neighbours

would help me if needed

1. I have social or leisure activities/ hobbies that I enjoy doing
2. I try to stay involved with things
3. I am healthy enough to have my independence
4. I can please myself what I do
5. I feel safe where I live
6. I get pleasure from my home
7. I take life as it comes and make the best of things
8. I feel lucky compared to most people
9. I have enough money to pay for household bills

**Geriatric Depression Scale (GDS-15, Short Form)**

***Instructions:*** Choose the best answer for how you felt over the past week. Note: when asking the patient to complete the form, provide the self-rated form (included on the following page).

| **No.** | **Question** | **Answer** | **Score** |
| --- | --- | --- | --- |
| 1. | Are you basically satisfied with your life? | YES / ***NO*** |  |
| 2. | Have you dropped many of your activities and interests? | ***YES*** / NO |  |
| 3. | Do you feel that your life is empty? | ***YES*** / NO |  |
| 4. | Do you often get bored? | ***YES*** / NO |  |
| 5. | Are you in good spirits most of the time? | YES / ***NO*** |  |
| 6. | Are you afraid that something bad is going to happen to you? | ***YES*** / NO |  |
| 7. | Do you feel happy most of the time? | YES / ***NO*** |  |
| 8. | Do you often feel helpless? | ***YES*** / NO |  |
| 9. | Do you prefer to stay at home, rather than going out and doing new things? | ***YES*** / NO |  |
| 10. | Do you feel you have more problems with memory than most people? | ***YES*** / NO |  |
| 11. | Do you think it is wonderful to be alive? | YES / ***NO*** |  |
| 12. | Do you feel pretty worthless the way you are now? | ***YES*** / NO |  |
| 13. | Do you feel full of energy? | YES / ***NO*** |  |
| 14. | Do you feel that your situation is hopeless? | ***YES*** / NO |  |
| 15. | Do you think that most people are better off than you are? | ***YES*** / NO |  |
| TOTAL | | |  |
