# Supplementary material for: Turkish validation of a resilience scale from older people’s perspectives: resilience scale for older adults
Source: PeerJ. 2025 Jan 21;13:e18837. doi: 10.7717/peerj.18837 (PMC11758914; doi:10.7717/peerj.18837)
Supplement: Supplemental Information 2 [file peerj-13-18837-s002.docx]

# **SOSYODEMOGRAFİK FORM**

**Cinsiyet:** Kadın/Erkek

**Yaş:** ….

**Medeni Durum:** Evli/Dul/Bekar

**Eğitim Seviyesi:** İlkokul / Ortaokul / Lise / Üniversite

**Yaşam Durumu:** Yalnız / Akraba veya eş ile bir evde / Huzurevinde / Bakıcı ile

**Gelir Durumu:** Gelirim giderimden az / Gelirim giderimle eşit / Gelirim giderimden fazla

**Çalışma Durumu:** Emekli / Halen çalışıyor (Yarı zamanlı/Tam zamanlı) / Hiç çalışmadı

**Meslek:** ….

**Kronik Hastalık Var mı?:** Evet/Hayır

**Kronik Hastalık Sayısı:** …

**Kronik Hastalıkları:** …

**Günlük Kullanılan İlaç Sayısı:** …

**Uyku Kalitesi:** İyi / Orta / Kötü

**Yaşlı Erişkinler İçin Dayanıklılık Ölçeği**

Aşağıda katılıp katılmadığınız bir dizi ifade yer almaktadır. Her bir madde için katılım derecenizi 1-5 ölçeği ile belirtiniz. Doğru veya yanlış cevap yoktur, bu nedenle açık ve dürüst olunuz.

| **Kesinlikle Katılmıyorum** | **Katılmıyorum** | **Kararsızım** | **Katılıyorum** | **Kesinlikle Katılıyorum** |
| --- | --- | --- | --- | --- |
| **1** | **2** | **3** | **4** | **5** |

1.Sıkıntılarla karşılaştığımda, azimle yoluma devam ederim.

2. Hedeflerime ulaşmakta kararlıyımdır.

3. Sırf zor diye bir şeyden vazgeçmem.

4. Bir işi kafama koyduğumda, onu layıkıyla yaparım.

5.Hedeflerime ulaşabilirim.

6. İşleri kendi başıma başarabilirim.

7.Genellikle sonunda her şeyin yoluna gireceğine inanırım.

8.Her durumdan en iyi şekilde faydalanmaya çalışırım.

9. Çoğu şeye karşı olumlu bir tutumum vardır.

10. Hayatıma anlam katan şeylerin farkındayım.

11. Hayatı dolu dolu yaşamaya çalışıyorum.

12.Her günü hayatımın son günüymüş gibi yaşamaya çalışırım.

13. Aidiyet hissim vardır (örn; aileme, arkadaş grubuma vs).

14. Yalnız değilim.

15. Eğer uzaklara gitseydim, insanlar beni özlerdi.

16. Güvenebileceğim aile fertlerim vardır.

17. Kötü bir şey olursa, ailemden yardım isteyebilirim.

18. Ailem için önemli olduğumu hissederim.

19. İhtiyacım olduğunda, arkadaşlarım yanımda olur.

20. Arkadaşlarım benim önemli destekçilerimdir.

21. İhtiyacım olduğunda komşularım bana yardım ederler.

22. Zor zamanlarımda yardım için dua ederim.

23. Kötü bir şey olduğunda, Allah’a dua etmek bununla baş etmemi sağlar.

24. Düzenli olarak dua ederim.

25. Hayat zorlaştığında Allah’a güvenirim.

26. Allah’ın beni koruduğuna inanırım.

27. Allah’ın dayanabileceğimden daha fazlasını vermeyeceğine inanırım.

28. Geçmiş deneyimlerimden çok şey öğrendim.

29. Hayatımda zorluklarla karşılaştım.

30.Karşılaştığım zorluklardan önemli dersler çıkardım.

31. İnsanın kendine iyi bakması önemlidir.

32.Kötü şeylerin olmasını önlemeye çalışırım.

33.Zorluklarla karşılaştığımda çözmeye çalışırım.

**Algılanan Stres Ölçeği (ASÖ)**

| **Notlar:**  YYK-Kısa ölçeği 13 maddeden oluşmaktadır, ayrıca genel yaşam kalitesi üzerine aşağıda görüldüğü gibi tek maddeli ön sorusu bulunmaktadır. Bu tek madde YYK ile skorlanmaz, Çok iyi’den (5) Çok kötü’ye (1) şeklinde kodlanır.  **YYK-Kısa skorlama:**  13 maddenin her biri Kesinlikle katılıyorum=5, Katılıyorum=4, Karasızım=3, Katılmıyorum=2, Kesinlikle Katılmıyorum=1 şeklinde skorlanır. Maddeler toplam YYK-kısa skoru için toplanır, böylece daha yüksek skorlar daha yüksek yaşam kalitesini gösterir. |
| --- |

**Yönerge:** Aşağıda geçtiğimiz ay içerisindeki kişisel deneyimleriniz hakkında bir dizi soru yöneltilmektedir. Her soruyu dikkatlice okuyarak size en uygun seçeneğin altındaki kutuya bir çarpı işareti koyarak cevaplayınız. Soruların doğru veya yanlış cevabı yoktur. Önemli olan sizin duygu ve düşüncelerinizi yansıtan yanıtları vermenizdir

Hiçbir Zaman; Neredeyse Hiçbir Zaman; Bazen; Oldukça Sık; Çok sık

1. Geçen ay, hayatınızdaki önemli şeyleri kontrol edemediğinizi ne sıklıkta hissettiniz?

2. Geçen ay, kişisel sorunlarınızı ele alma yeteneğinize ne sıklıkta güven duydunuz?

3. Geçen ay, her şeyin yolunda gittiğini ne sıklıkta hissettiniz?

4. Geçen ay, ne sıklıkta problemlerin üstesinden gelemeyeceğiniz kadar biriktiğini hissettiniz

**Yaşlılarda Yaşam Kalitesi Ölçeği (YYK-Kısa)**

**Size yaşam kaliteniz hakkında sorular sormak istiyoruz:**

Tek madde-genel YK:

1. **Yaşam kalitenizi oluşturan iyi ve kötü şeyleri düşündüğünüzde, bir bütün olarak yaşam kalitenizi nasıl derecelendirirsiniz?**

Bütün olarak yaşam kaliteniz:

- Çok iyi
- İyi
- Fena değil
- Kötü
- Çok kötü

**YYK-Kısa**

**2) Lütfen her soruda bir kutuyu işaretleyiniz. Lütfen sizi/görüşlerinizi en iyi tanımlayan cevabı seçiniz. Doğru veya yanlış cevap yoktur.**

|  | Kesinlikle katılıyorum | Katılıyorum | Kararsızım | Katılmıyorum | Kesinlikle katılmıyorum |
| --- | --- | --- | --- | --- | --- |
| 1- Genel olarak yaşamımdan keyif alırım. |  |  |  |  |  |
| 2- Olmasını sabırsızlıkla beklediğim şeyler var. |  |  |  |  |  |
| 3- Evden dışarı çıkıp dolaşmak için yeterince sağlıklıyım . |  |  |  |  |  |
| 4- Ailem, arkadaşlarım veya komşularım ihtiyaç olursa bana yardım ederler. |  |  |  |  |  |
| 5- Yapmaktan keyif aldığım sosyal veya boş zaman aktivitelerim/hobilerim var. |  |  |  |  |  |
| 6- Bir şeylerle meşgul olmaya çalışıyorum. |  |  |  |  |  |
| 7- Bağımsızlığıma sahip olmak için yeterince sağlıklıyım. |  |  |  |  |  |
| 8- Yaptıklarımla kendimi memnun edebiliyorum. |  |  |  |  |  |
| 9- Yaşadığım yerde kendimi güvende hissediyorum. |  |  |  |  |  |
| 10- Evimden keyif alıyorum. |  |  |  |  |  |
| 11- Hayatı olduğu gibi kabul ediyorum ve en iyi şekilde değerlendiriyorum. |  |  |  |  |  |
| 12- Çoğu insana göre kendimi şanslı hissediyorum. |  |  |  |  |  |
| 13- Ev faturalarını ödemek için yeterince param var. |  |  |  |  |  |

**YESAVAGE GERİATRİK DEPRESYON SKALASI**

Geçtiğimiz 7 gün boyunca nasıl hissettiğinizi en iyi tanımlayan EVET ya da HAYIR cevabını veriniz.

|  | **Evet** | **Hayır** |
| --- | --- | --- |
| 1. Genel olarak hayatınızdan memnun musunuz? |  |  |
| 2. Aktivitelerinizin ve ilgi alanlarınızın çoğundan uzaklaştınız mı? |  |  |
| 3. Hayatınızın boş/anlamsız olduğunu düşünüyor musunuz? |  |  |
| 4. Çoğunlukla canınız sıkılır mı? |  |  |
| 5. Çoğu zaman moraliniz iyi midir? |  |  |
| 6. Başınıza kötü bir şey geleceğinden korkuyor musunuz? Kendinize kötü bir şeyler olacağını düşünerek korkar mısınız? |  |  |
| 7. Çoğunlukla kendinizi mutlu hissediyor musunuz? |  |  |
| 8. Sıklıkla kendinizi çaresiz/yardıma muhtaç hissediyor musunuz? |  |  |
| 9. Dışarı çıkmak ve yeni şeyler yapmak yerine evde oturmayı tercih ediyor musunuz? |  |  |
| 10. Hafızanızla ilgili olarak çoğu kişiden daha fazla probleminiz olduğunu düşünüyor musunuz? |  |  |
| 11. Şu anda yaşıyor olmanın güzel bir şey olduğunu düşünüyor musunuz? |  |  |
| 12. Son zamanlarda kendinizi değersiz hissediyor musunuz? |  |  |
| 13. Kendinizi enerji dolu hissediyor musunuz? |  |  |
| 14. Durumunuzun ümitsiz olduğunu düşünüyor musunuz? |  |  |
| 15. Çoğu kişinin sizden daha iyi durumda olduğunu düşünüyor musunuz? |  |  |
